# Gastrointestinal *Mycobacterium avium* complex in advanced HIV: insights from advanced endoscopic imaging and review of endoscopic features

**DOI:** 10.1016/j.vgie.2025.11.006

**Published:** 2025-11-24

**Authors:** Mohammed Adnan Khan, Rima Tayfour, Ahmed Al-Salami, Halima Al Shuaili, Sinan Sharba, Kawthar Al amri, Nelly Kanberg, Samer Al-Dury

**Affiliations:** 1 Muscat Endoscopy Academy, Endoscopy Unit, Department of Gastroenterology, Medical City Hospital for Military and Security Services, Muscat, Oman; 2 Darlington Memorial Hospital, County Durham and Darlington NHS Foundation Trust, Darlington, UK; 3 Department of Pathology, Medical City Hospital for Military and Security Services, Muscat, Oman; 4 Sahlgrenska Academy, Institute of Medicine, University of Gothenburg, Gothenburg, Sweden; 5 Department of Infectious Diseases, Medical City Hospital for Military and Security Services, Muscat, Oman; 6 Department of Infectious Diseases, Institute of Biomedicine, University of Gothenburg, Gothenburg, Sweden

## Abstract

**Background and Aims:**

Gastrointestinal (GI) *Mycobacterium avium* complex (MAC) infection remains an under-recognized adverse event in severely immunocompromised patients. We present a case in which advanced upper GI endoscopy and high-resolution capsule endoscopy together provided unprecedented visualization of disease progression, forming the basis for a structured diagnostic review.

**Methods:**

A 21-year-old woman with HIV (cluster of differentiation 4 count 8/μL) presented with chronic diarrhea, weight loss, and hypoalbuminemia. Upper endoscopy revealed severe duodenal villous atrophy and mucosal scalloping. Subsequent capsule endoscopy captured progressive mucosal changes extending into the distal jejunum and ileum, including pseudo-Whipple's disease nodularity. Diagnosis was confirmed by histology and polymerase chain reaction. A systematic review of 18 adult GI MAC cases with endoscopic documentation was performed to identify consistent visual patterns.

**Results:**

Multimodal imaging revealed a continuum of pathology not previously captured in this detail. Literature synthesis showed that duodenojejunal nodularity, villous atrophy, and scalloping were the most common findings. On the basis of these patterns, we propose a 9-point MAC Suspicion Score to prompt early diagnostic consideration in high-risk patients.

**Conclusions:**

This case exemplifies how advanced endoscopic imaging can reveal subtle but diagnostic features of GI MAC and support a structured recognition strategy in severely immunocompromised hosts.

## Introduction

*Mycobacterium avium* complex (MAC) remains a significant opportunistic infection in individuals with advanced HIV infection, particularly in those with poor adherence to antiretroviral therapy (ART).^1^ Although disseminated MAC has become less common in the ART era, gastrointestinal (GI) involvement continues to occur, especially when cluster of differentiation 4 (CD4) positive T lymphocyte T-cell counts fall below 50 cells/μL.^1^ The organisms, acquired via ingestion or inhalation, can disseminate hematogenously in immunocompromised hosts.^2^ Although GI MAC most commonly affects individuals with advanced HIV/AIDS, other at-risk populations include patients receiving biologic or prolonged immunosuppressive therapy, solid-organ or stem-cell transplant recipients, individuals with hematologic malignancies, and those with primary immunodeficiency disorders.

GI MAC is often under-recognized because of nonspecific symptoms—diarrhea, abdominal pain, weight loss, and fever—which overlap with other infections and malabsorptive syndromes in patients with AIDS.^2^^,^^3^ The small intestine, particularly the duodenum and proximal jejunum, is the most frequently affected site.^2^^,^^3^ Endoscopic findings are variable; nodules, plaques, villous atrophy, and granular or edematous mucosa have been reported, but some patients show no visible lesions.^4^^,^^5^ Histologically, MAC infection is characterized by infiltration of the lamina propria with lipid-laden macrophages containing acid-fast bacilli (AFB), typically without granuloma formation.^4^^,^^5^

**Figure 1:**
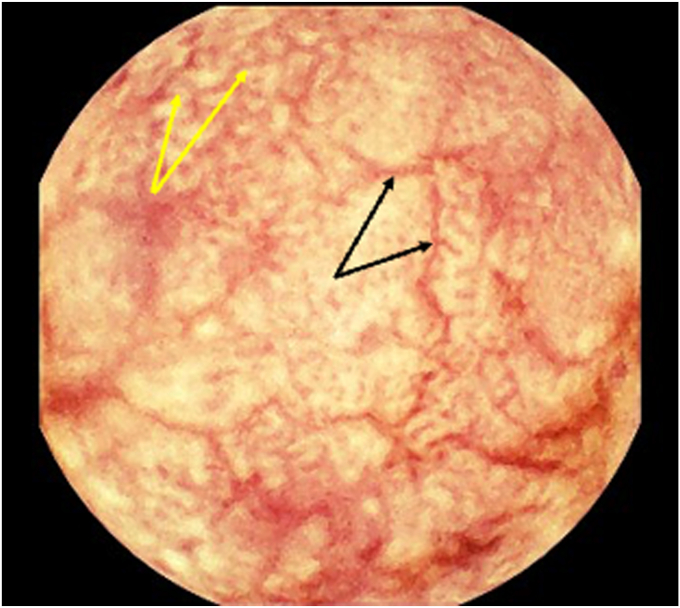
Proximal jejunum with diffuse villous blunting (*yellow arrows*), scalloping, and granular mosaic pattern consistent with severe mucosal atrophy (*black arrows*).

Because of the protean presentation and patchy involvement, diagnosis often requires a high index of suspicion and targeted intestinal biopsies with AFB staining, culture, or polymerase chain reaction (PCR). Capsule endoscopy may be particularly useful in revealing subtle or extensive small-bowel involvement not seen on routine esophagogastroduodenoscopy (EGD) or colonoscopy (Fig. 1).

We present a case of GI MAC in a young woman with AIDS, showcasing high-resolution endoscopic and video capsule imaging. The visual documentation captured here provides rare details of disease progression along the small intestine. We also review published cases and propose a structured clinical-endoscopic score to aid in early recognition of GI MAC in high-risk patients.

## Case presentation

A 21-year-old woman with a history of HIV and longstanding poor adherence to ART was admitted with a 3-month history of progressive watery diarrhea, unintentional weight loss (approximately 15 kg), intermittent low-grade fevers, and diffuse abdominal discomfort. She reported multiple daily episodes of nonbloody diarrhea and dull, generalized abdominal pain. On physical examination, she appeared cachectic with temporal wasting, mild abdominal distension, and diffuse tenderness without peritonism. There was no peripheral lymphadenopathy or organomegaly.

Laboratory testing revealed profound immunosuppression with a CD4 positive T-cell count of 8 cells/mm^3^ and HIV-1 viral load exceeding 1,000,000 copies/mL. Additional laboratory testing showed anemia (hemoglobin level: 8.5 g/dL), hypoalbuminemia (albumin level: 20 g/L), and elevated inflammatory markers (C-reactive protein level: 80 mg/L). Stool testing results for bacterial and parasitic pathogens, including *Clostridioides difficile*, *Salmonella*, *Shigella*, *Campylobacter*, and *Giardia*, were negative. Serum cytomegalovirus (CMV) PCR results were intermittently positive, but CMV was not detected in GI tissue or aspirates.

**Figure 2:**
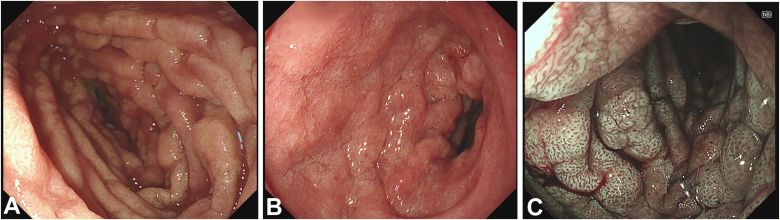
Duodenal mucosa on esophagogastroduodenoscopy demonstrating diffuse villous injury. **A,** White-light endoscopy showing pale, edematous duodenal folds with marked villous blunting. **B,** Closer white-light view demonstrating nodular, edematous mucosa with loss of normal villous architecture. **C,** Narrow-band imaging highlighting a mosaic mucosal pattern and villous distortion consistent with diffuse mucosal involvement.

Because of persistent GI symptoms, upper endoscopy (EGD) and colonoscopy were performed. EGD showed mildly erythematous gastric mucosa and a diffusely edematous, pale duodenum with markedly blunted villi. Narrow-band imaging (NBI) enhanced the visualization of these duodenal changes (Fig. 2). Push enteroscopy revealed similar findings extending into the proximal jejunum, although the distal extent of disease remained unclear (Video 1, available online at www.videogie.org). Colonoscopy was unremarkable both macroscopically and histologically.

**Figure 3:**
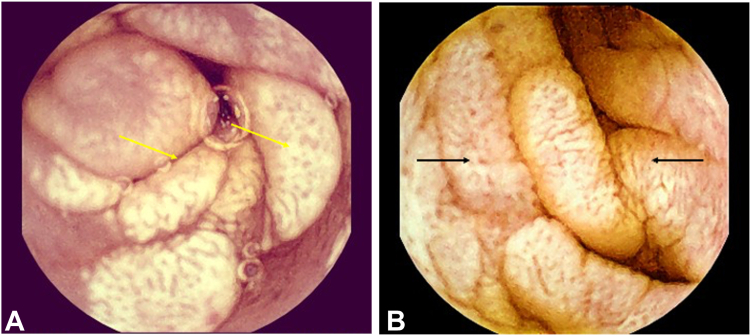
High-resolution capsule endoscopy images showing the progression of small-bowel mucosal changes in a patient with gastrointestinal *Mycobacterium avium* complex (MAC) infection, arranged from proximal (**A**) to distal (**B**) segments. The findings reflect the evolving phenotype of MAC enteritis along the small bowel, from atrophic to infiltrative noduloplaque lesions. **A,** Mid-to-distal jejunum demonstrating nodular mucosa with white-yellow plaque-like elevations resembling a pseudo-Whipple's disease appearance (*yellow arrows*). **B,** Distal ileum showing circumferential mucosal thickening, coarse nodularity, and patchy mucosal loss (*black arrows*).

Given ongoing diarrhea, malnutrition, and diagnostic uncertainty, capsule endoscopy was pursued using the MiroCam (IntroMedic, Seoul, South Korea) system. The study revealed striking abnormalities throughout the jejunum and ileum. The mucosa appeared diffusely scalloped with a patchy mosaic pattern and granular surface (Figs. 1 and 3), consistent with severe small-bowel enteritis. Several areas of villous flattening, whitish nodularity, and mucosal atrophy were also seen. A representative 1-minute video excerpt from the capsule study is available in Video 2, available online at www.videogie.org.

**Figure 4:**
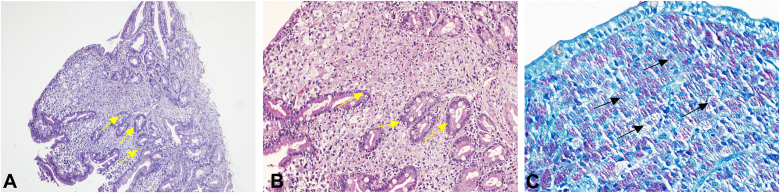
Duodenal mucosa with *Mycobacterium avium* complex. **A and B,** Duodenal mucosa with lamina propria infiltrated by sheets of histiocytes with abundant cytoplasm mixed with lymphocytes and plasma cells (*yellow arrows*) (H&E, ×100 and ×200). **C,** Ziehl-Neelsen (ZN) stain demonstrates numerous slender, beaded, acid-fast bacilli within the histiocytes (*black arrows*) (ZN, ×400).

Histologic analysis of duodenal and jejunal biopsies showed expansion of the lamina propria by sheets of foamy histiocytes (Fig. 4A), which stained positively for AFB (Ziehl-Neelsen, Fig. 4B) and for CD68, confirming macrophage origin. Periodic acid–Schiff (PAS) staining results was also positive. Tissue PCR results were positive for MAC. Stomach biopsy results were similarly positive for MAC; colonic tissue was normal. No evidence of *Cryptosporidium*, CMV, *Mycobacterium tuberculosis*, or other coinfections was found in tissue or stool samples.

The diagnosis of disseminated MAC infection with prominent GI involvement and protein-losing enteropathy was established. Initial treatment with oral antimycobacterial therapy (clarithromycin, ethambutol, rifampicin, and amikacin) was limited by concerns of poor drug absorption due to mucosal damage. Intravenous therapy was initiated, along with intravenous albumin and a high-protein, semielemental enteral diet. Clinical improvement followed within 2 weeks, with resolution of diarrhea, gradual weight gain, and improved serum albumin levels, reaching 28 g/L. ART was reinitiated under close monitoring for immune reconstitution inflammatory syndrome. Plans were made for follow-up endoscopy to assess mucosal healing 3 to 6 months after MAC therapy; however, after initial improvement of the patient's GI issues/malnutrition, her clinical course became complicated by intercurrent opportunistic infections and psychosocial challenges associated with ART interruption. In consultation with the treating team, we considered that proceeding with an elective control endoscopy was inappropriate on clinical and ethical grounds, and further research procedures were not pursued.

## Methods

### Literature search strategy

We conducted a focused literature review to identify reported cases of GI MAC infection in adult patients, with an emphasis on endoscopic findings. PubMed, MEDLINE, Embase, Cochrane Library, and Scopus were searched for articles published between January 1988 and March 2025. Search terms included “Mycobacterium avium complex” OR “MAC” AND “gastrointestinal” OR “enteritis” OR “small bowel” OR “duodenum” OR “jejunum” AND “endoscopy” OR “capsule enteroscopy” OR “video capsule endoscopy” OR “push enteroscopy” OR “colonoscopy’’ AND “case” OR “case report” OR “case series.” Reference lists of relevant articles were also manually screened for additional eligible studies.

### Inclusion and exclusion criteria

Studies were included if they:

• Reported adult patients (aged ≥18 years) with confirmed GI MAC infection diagnosed by histology (acid-fast staining), culture, or PCR; and
• Described GI endoscopic findings via EGD, colonoscopy, push enteroscopy, or capsule endoscopy.

The exclusion criteria were as follows:

• Pediatric cases.
• Reports lacking endoscopic descriptions or images.
• Abstracts, correspondence, or reviews without individual patient-level data.

### Data extraction and analysis

From each eligible study, the following variables were extracted:

• Demographics: age, sex, and immune status (CD4 count or transplant/immunosuppressive therapy).
• Clinical features: GI symptoms, symptom duration, and weight loss.
• Diagnostic findings: endoscopic appearance, anatomical site of involvement, and histologic confirmation.
• Therapy and outcomes, if available.

Endoscopic findings were categorized based on macroscopic mucosal patterns, including nodularity, plaques, villous atrophy, scalloping, ulceration, and granularity. These features were synthesized to identify recurring patterns across cases.

## Results

Our systematic review identified 18 adult cases of GI MAC infection with documented endoscopic findings, published between 1988 and 2025. These reports included both individual case studies and small series, all meeting inclusion criteria of confirmed MAC by histology, culture, or PCR, along with described or imaged endoscopic appearances.

The median patient age was 38 years (range: 21-76 years), with approximately 75% being male. Most patients (15 of 18, 83%) were diagnosed with advanced AIDS and had CD4 counts below 50 cells/μL. The remaining 3 cases involved non-HIV immunosuppression, including organ transplant recipients. The most common presenting symptoms were chronic diarrhea (89%), weight loss (78%), fever (56%), and abdominal pain (50%).

Anatomically, the duodenum and proximal jejunum were the most frequently affected segments, involved in 83% of cases. Involvement of the ileum, colon, or rectum occurred in 28% of cases. In patients who underwent capsule endoscopy, abnormalities were often diffuse and extended throughout the small bowel.

Endoscopic features varied in appearance and distribution but followed consistent themes. Whitish or yellowish mucosal nodules or plaques were the most frequently reported finding (72%), often appearing scattered or diffusely distributed in the duodenum and jejunum. Villous blunting or atrophy was observed in 56% of cases, followed by mucosal scalloping or a mosaic-like pattern (39%), reminiscent of celiac disease. Granular mucosa—typically coarse or nodular—was seen in 33%, whereas shallow ulcerations or erosions were noted in 28%. Notably, 3 cases (17%) had macroscopically normal mucosa on endoscopy, despite histologic confirmation of MAC, highlighting the potential for diagnostic delay or under-recognition.

A detailed overview of each case—including immune status, presenting symptoms, site of involvement, and mucosal findings—is summarized in Table 1. On the basis of the review, we developed a practical 9-point clinical-endoscopic risk score (MAC Suspicion Score [MAC-SS]) to guide early consideration of GI MAC in immunocompromised patients. This score is intended to aid bedside assessment and decision-making regarding biopsy and targeted mycobacterial workup, particularly when endoscopic appearances are subtle or nonspecific.

**Table 1:** Summary of published adult cases of GI *Mycobacterium avium* complex infection with endoscopic findings (1988-2025)

Author (year)	Patient demographics and immune status	GI symptoms	Site of GI involvement	Endoscopic findings
Vázquez-Iglesias et al (1988)^6^	24-year-old woman with AIDS (CD4 not reported)	Chronic diarrhea, weight loss; abdominal lymphadenopathy	Duodenum (second portion)	Multiple yellow-white nodules replacing mucosa, initial description of MAC duodenitis
Gray and Rabeneck (1989)^7^	35 homosexual men with advanced AIDS (CD4 likely <100)	Chronic diarrhea, weight loss, malabsorption	Duodenum (most), rectum,^8^ esophagus	Often normal or mildly abnormal; 12 with fine mucosal granularity
Monsour et al (1991)^9^	2 adult men with AIDS	Chronic diarrhea, weight loss	Duodenum (second part)	Numerous 2- to 4-mm nodules with cobblestone appearance, AFB+ macrophages
Poorman and Katon (1994)^10^	34-year-old man with AIDS	Chronic diarrhea, weight loss	Duodenum, jejunum	Whitish nodular mucosa mimicking Whipple's disease; MAC confirmed by biopsy
Cappell and Philogene (1995)^11^	31-year-old man with advanced AIDS	Chronic diarrhea, GI bleeding, anemia	Duodenum (descending part)	Coarsely granular mucosa due to MAC villous engorgement
Sun et al (2005)^12^	38-year-old man with AIDS (CD4 <50)	Chronic diarrhea, abdominal pain, fever, weight loss	Duodenum (primary site)	Granular, erythematous mucosa with ulcers
Dray et al (2007)^13^	40-year-old man with AIDS	Diarrhea, weight loss	Duodenum	Whitish plaques resembling Whipple's disease, AFB+ on biopsy
Yamada et al (2011)^8^	42-year-old man with AIDS (CD4 10)	Abdominal pain, chronic diarrhea	Entire small intestine	Capsule: multiple whitish nodules; AFB+ granulomas, stool MAC+
Vaz et al (2017)^3^	25-year-old man with AIDS (CD4 approximately 34)	Abdominal pain, fever, vomiting, diarrhea (10 mo)	Proximal > distal small intestine	VCE: edema, lymphangiectasia, ulcers; duodenal biopsy MAC+
Al-Shammari et al (2017)^14^	27-year-old man with AIDS (CD4 6)	Severe diarrhea, septic state	Colon (rectosigmoid)	Flat whitish nonulcerative plaques; AFB+ macrophages
Rezaie et al (2019)^15^	31-year-old man with AIDS (CD4 38)	Rectal pain, diarrhea, chills	Colon (rectum)	Single rectal erosion only; biopsy showed AFB+
Ingilizova et al (2019)^16^	56-year-old man, renal transplant, immunosuppressed	Diarrhea, weight loss, protein-losing enteropathy	Colon	No detailed lesions; biopsy showed MAC in submucosa
Chirayath et al (2021)^17^	37-year-old man with AIDS	Abdominal pain, weight loss	Duodenum	White plaque-like mucosa, villous blunting; MAC on PCR/culture
Mita et al (2025)^18^	76-year-old woman, liver transplant, prior pulmonary MAC	Fever, vomiting, small-bowel obstruction	Jejunum (surgical anastomosis)	Mass-like ulcerated lesion at anastomosis; AFB+ histiocytes

All patients were severely immunocompromised.

*AFB*, Acid-fast bacilli; *CD4*, cluster of differentiation 4; *GI*, gastrointestinal; *MAC*, *Mycobacterium avium* complex; *PCR*, polymerase chain reaction; *VCE*, video capsule endoscopy.

In our index case, the progression of mucosal features—from subtle villous atrophy and scalloping on upper endoscopy to cerebriform plaque-like nodules in the distal small bowel—mirrored patterns described in the literature. High-resolution endoscopic and video capsule imaging allowed for detailed visualization of these evolving patterns, adding to the spectrum of documented appearances of GI MAC and supporting the broader applicability of the derived MAC-SS.

## Discussion

This case highlights several important insights into the endoscopic presentation and diagnostic challenges of GI MAC infection in immunocompromised patients, particularly those with advanced HIV/AIDS. Although most cases occur in advanced HIV/AIDS, gastrointestinal MAC may also affect other immunocompromised hosts, including transplant recipients, patients on biologic or prolonged immunosuppressive therapy, individuals with hematologic malignancies, and those with primary immunodeficiency disorders.

First, it demonstrates that GI MAC can present with evolving, segmentally progressive mucosal changes—from subtle proximal villous atrophy to overt nodular infiltration distally. In our patient, initial EGD and push enteroscopy revealed severe duodenal villous blunting, mucosal scalloping, and a granular “mosaic” pattern without obvious plaques. These changes likely reflect early-phase infiltration of the lamina propria by lipid-laden histiocytes. As the disease progressed distally, capsule endoscopy revealed increasingly striking features—dense, cerebriform mucosa with raised whitish nodules and plaques consistent with a pseudo-Whipple's disease–like appearance. This transition, visualized in high resolution from jejunum to ileum, has not been previously documented in such detail, to our knowledge, underscoring the utility of capsule imaging in mapping mucosal transformation over distance and time.

Second, the case reinforces the role of advanced endoscopic imaging—both conventional and capsule-based—in detecting subtle and varied manifestations of GI MAC. In several published reports, endoscopic findings were minimal or absent despite significant disease on histology.^2^^,^^15^ Similarly, Mita et al^18^ reported a case in which standard EGD appeared normal, and capsule endoscopy ultimately revealed the pathology. Our case supports this sequence and emphasizes that when mucosal abnormalities are seen, they may be nonspecific and depend on disease location and phase. NBI during EGD in our case also enhanced visualization of villous loss and edema, reinforcing the added value of advanced optics even in routine procedures.

Third, this case illustrates the common diagnostic delays associated with GI MAC. The patient's symptoms—chronic diarrhea, weight loss, and hypoalbuminemia—were initially attributed to malnutrition, CMV, and other comorbidities, leading to a delay in definitive diagnosis and targeted therapy. This reflects a pattern seen across the literature, where GI MAC is often only considered after conventional causes have been excluded. In our review, nearly one-third of cases reported normal or nonspecific endoscopic findings, and diagnosis was frequently made only after biopsy and mycobacterial studies.

To facilitate earlier suspicion and intervention, we propose a practical clinical-endoscopic risk score—MAC-SS. The 9-point tool assigns 1 point for each of the following: (1) profound immunosuppression (eg, CD4 <50), (2) chronic GI symptoms, (3) weight loss or malnutrition, (4) hypoalbuminemia, (5) mucosal plaques or nodules, (6) diffuse villous atrophy or edema, (7) mucosal scalloping, (8) shallow ulcers or erosions, and (9) granular or mosaic mucosa (Table 2). Although not diagnostic, a score ≥3 may prompt targeted duodenal/jejunal biopsies with AFB staining, mycobacterial culture, and/or PCR. Because item prevalence is heterogeneous—with some findings present in only a small subset of cases—the MAC-SS should be interpreted as a composite signal rather than any pathognomonic marker, with thresholds to be refined by prospective validation.

**Table 2:** Clinical Suspicion Score for GI MAC Suspicion Score (1 point per feature)

Feature	Description
Immunosuppression	CD4 <50 or post-transplant immunosuppression
Chronic GI symptoms	Prolonged diarrhea, abdominal pain
Weight loss or malnutrition	Significant cachexia or body mass index loss
Hypoalbuminemia	Suggestive of protein-losing enteropathy
Mucosal plaques/nodules	Seen on esophagogastroduodenoscopy, enteroscopy, or capsule endoscopy
Villous atrophy or edema	Blunted folds or diffuse mucosal swelling
Mucosal scalloping	Scalloped duodenal or jejunal folds
Shallow ulcers or erosions	Noncratered mucosal breaks
Granular or mosaic mucosa	“Cobblestone” or patchy surface patterns

A score ≥3 may prompt biopsy and microbiologic testing.

*GI*, Gastrointestinal; *MAC*, *Mycobacterium avium* complex.

MAC-SS should be applied with clinical judgment because several included features are nonspecific and overlap with mimics (eg, celiac disease, Whipple's disease, CMV enteritis, and intestinal lymphoma). Scalloping/mosaicism are classically seen in celiac disease, whereas plaques/nodules may occur in Whipple's disease and CMV; therefore, histologic and microbiologic confirmation remains essential. MAC-SS is a pragmatic, uniformly weighted (1-point/item) awareness tool intended for immunocompromised hosts and not for use outside this context. Prospective validation is required to define operating characteristics (sensitivity, specificity, and inter-rater reliability) and an optimal weighting of the presented features. With additional cases, we will explore optimal combinations of symptoms and endoscopic findings to enhance clinical usability.

When MAC is suspected, we suggest to target D2 and proximal jejunum; aim to obtain ≥6 biopsies when feasible; request Ziehl-Neelsen (AFB), PAS, CD68; and order mycobacterial PCR/culture. Consider additional/deeper samples if the mucosa appears normal. Of note, specimens must be placed in either formalin or pure saline solution (for histology and PCR, respectively) before immediate transportation to the laboratory for further processing.

Clinical treatment response in our patient was favorable after initiation of combination antimycobacterial therapy (rifampicin, ethambutol, clarithromycin, and amikacin) and nutritional support. Because of concerns over impaired absorption in the setting of diffuse enteropathy, intravenous therapy was initially required. This highlights another clinical implication: when MAC causes severe mucosal disruption, oral therapy may be insufficient. Early diagnosis allows for timely adjustment of delivery routes and avoids adverse events such as cachexia, protein-losing enteropathy, or bowel obstruction.

Finally, the imaging quality in this case represents a state-of-the-art demonstration of endoscopic features in GI MAC. The sequential capsule stills and video montage allow readers to appreciate not only specific findings (scalloping, mosaicism, and nodules), but also their evolution along the bowel. These images may serve as a valuable teaching and reference tool, particularly for endoscopists evaluating immunocompromised patients.

## Patient Consent

The patient provided verbal and written consent to participating in this study.

## Disclosure

All authors disclosed no financial relationships.

## Supplementary data

Video Narrated overview of the endoscopic, capsule endoscopy, and histopathologic features of gastrointestinal Mycobacterium avium complex infection, using the presented case to highlight characteristic findings and relevant patterns reported in the literature.

Video 1 Upper gastrointestinal in a patient with advanced HIV demonstrating duodenal and proximal jejunal mucosa with diffuse villous atrophy, blunted folds, and pale mucosal appearance. No focal ulcers or masses are seen. Findings are consistent with extensive mucosal infiltration in gastrointestinal *Mycobacterium avium* complex infection.

Video 2 One-minute capsule endoscopy clip showing progression of gastrointestinal *Mycobacterium avium* complex enteritis from proximal scalloping and mosaicism to distal nodular, plaque-like mucosa consistent with a pseudo-Whipple’ disease appearance.
